# A New Thermophilic Ene-Reductase from the Filamentous Anoxygenic Phototrophic Bacterium *Chloroflexus aggregans*

**DOI:** 10.3390/microorganisms9050953

**Published:** 2021-04-28

**Authors:** Marina Simona Robescu, Mattia Niero, Giovanni Loprete, Laura Cendron, Elisabetta Bergantino

**Affiliations:** Department of Biology, University of Padova, Viale G. Colombo 3, 35131 Padova, Italy; marinasimona.robescu@studenti.unipd.it (M.S.R.); mattia.niero87@gmail.com (M.N.); giovanni.loprete@studenti.unipd.it (G.L.)

**Keywords:** ene-reductases, photosynthetic extremophiles, thermophilic-like old yellow enzymes, *Chloroflexus aggregans*

## Abstract

Aiming at expanding the biocatalytic toolbox of ene-reductase enzymes, we decided to explore photosynthetic extremophile microorganisms as unique reservoir of (new) biocatalytic activities. We selected a new thermophilic ene-reductase homologue in *Chloroflexus aggregans*, a peculiar filamentous bacterium. We report here on the functional and structural characterization of this new enzyme, which we called *Ca*OYE. Produced in high yields in recombinant form, it proved to be a robust biocatalyst showing high thermostability, good solvent tolerance and a wide range of pH optimum. In a preliminary screening, *Ca*OYE displayed a restricted substrate spectrum (with generally lower activities compared to other ene-reductases); however, given the amazing metabolic ductility and versatility of *Chloroflexus aggregans*, further investigations could pinpoint peculiar chemical activities. X-ray crystal structure has been determined, revealing conserved features of Class III (or thermophilic-like group) of the family of Old Yellow Enzymes: in the crystal packing, the enzyme was found to assemble as dimer even if it behaves as a monomer in solution. The description of *Ca*OYE catalytic properties and crystal structure provides new details useful for enlarging knowledge, development and application of this class of enzymes.

## 1. Introduction

Microorganisms that live in extreme environments produce extremozymes, enzymes that have developed molecular mechanisms of adaptation to extreme physico-chemical conditions. Such properties make them very attractive as biocatalysts in industrial biotransformations [1], since industrial processes often require robust enzymes able to operate under challenging conditions such as high substrate concentration and temperature, farthest values of pH or salinity. In the last decade, therefore, attention to extreme environments has increased in the field of biocatalysis in order to find previously unknown extremophilic microorganisms to be exploited as mines for new chemical activities.

**Scheme 1:**
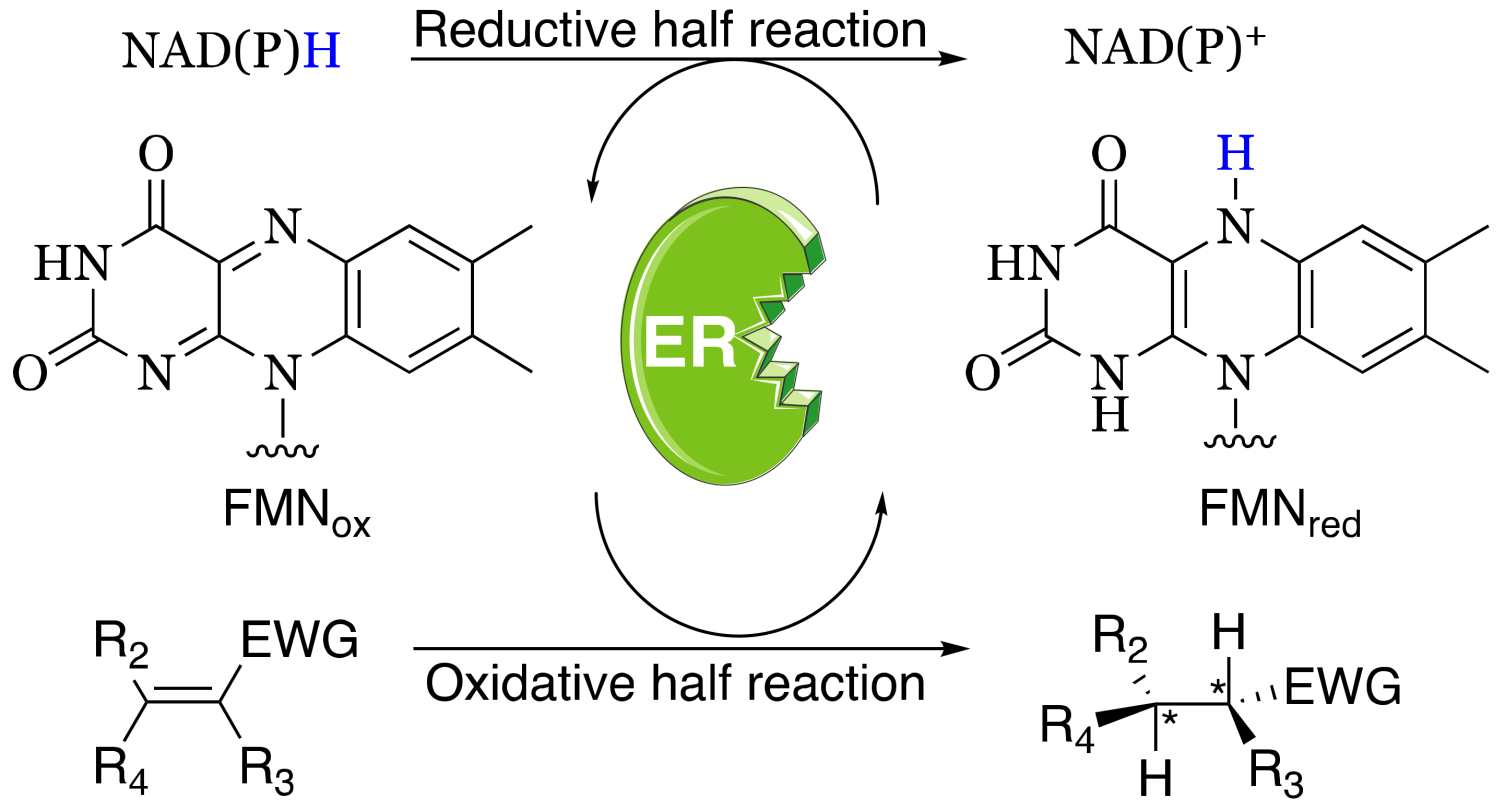
Stereoselective reduction of an activated C=C double bond catalyzed by ene-reductases (ERs). NAD(P)H acts as an electron donor to reduce the flavin cofactor FMN. EWG: electron withdrawing group.

Ene-reductases (ERs) from the family of the Old Yellow Enzyme (OYE; E.C. 1.6.99.1) are nicotinamide-dependent flavoproteins catalyzing the asymmetric hydrogenation of a wide panel of activated alkenes (e.g., α,β-unsaturated ketones, aldehydes, nitroalkenes, carboxylic acids, and derivatives) (Scheme 1). They are ubiquitous in nature and have been found in yeasts, bacteria, plants and parasitic eukaryotes [2]. Nevertheless, their physiological role is still not elucidated, apart from a few cases. They have been suggested to take part in the detoxification of electrophilic compounds, both endogenous and xenobiotic, and in the response to the oxidative stress [3]. Moreover, the involvement of some OYE-homologues in specialized metabolic pathways has been described in different organisms: jasmonic acid biosynthesis in plants [4], ergot alkaloid biosynthesis in *Aspergillus fumigatus* [5] and prostaglandin F_2α_ biosynthesis in *Trypanosoma cruzi* [6]. Despite the lack of knowledge regarding their roles as native enzymes, their assorted potentialities as biocatalysts have prompted the investigation by both academic and industrial researchers and, over the last years, the number of available ERs has grown significantly. However, the demand for new ERs keeps growing in order to expand the biocatalytic toolbox and to overcome some limitations still encountered in their application (low stability in the harsh industrial conditions, modest turnover numbers, poor substrate tolerance and, in some cases, low enantioselectivities) [7]. Furthermore, in recent years, unexpected reactivities due to the chemical versatility of flavin cofactor, have been reported for this class of enzymes [8,9,10,11,12,13,14]. So, the discovery of new ERs has moved far beyond the classical C=C-bond reductions, driven by curiosity to (completely) understand the potentialities of this class of biocatalysts.

The great majority of reported ERs are derived from bacterial, fungal and plant sources, and generally operate under mild reaction conditions [2]. A handful of ERs with improved thermostability has been isolated from extremophile organisms: TOYE from *Thermoanaerobacter pseudethanolicus* [15], chromate reductase from *Thermus scotoductus* SA-01 (*Ts*OYE) [16], *Gk*OYE from *Geobacillus kaustophilus* [17], *Geo*ER from *Geobacillus* sp. #30 [18], *Chr*OYE3 from *Chryseobacterium* sp. CA49 [19], FOYE-1 from the acidophilic iron-oxidizing bacterium *Ferrovum* sp. JA12 [20], *Ct*OYE from the thermophilic cyanobacterium *Chroococcidiopsis thermalis* and *Gs*OYE from the polyextremophilic alga *Galdieria sulphuraria* [21].

Aiming at increasing the protein diversity of this class of enzymes, by using as query the sequence of YqjM (from *Bacillus subtilis*) that is conventionally considered a representative member for the thermophilic-like subclasses of OYEs (Class II or Class III, following Peters et al. [22] or Böhmer et al. [23], respectively), we initiated a search for OYE homologues in unconventional organisms such as photosynthetic extremophile bacteria. To our knowledge, indeed, only higher plants [24,25,26], few examples of cyanobacteria [21,27,28] and two algae [21,23] have been explored so far as sources of ene-reductases. Among the putative enzymes identified, we selected one protein from *Chloroflexus aggregans*.

This microorganism is known as a thermophilic filamentous, anoxygenic and sulfide-dependent phototrophic bacterium belonging to the family of *Chloroflexaceae*. It was firstly isolated from bacterial mats in Yufuin and Meotobuchi hot springs in Japan [29], then found widely distributed in terrestrial hot springs, in the temperature range of 50–70 °C with an optimal temperature of growth of 55 °C. It is characterized by the ability to form multicellular aggregates endowed with gliding motility, in symbiosis with other organisms such as thermophilic cyanobacteria, sulphur-oxidizing bacteria and aerobic and anaerobic heterotrophic bacteria [30]. Recent studies have shown that, besides being able to grow photoeterotrophically under anaerobic conditions in the light and chemoeterotrophically under aerobic conditions in the dark using sulfide furnished by the symbiotic organisms, some isolates can thrive photoautotrophically in anaerobiosis and chemolithotrophically in aerobiosis using H_2_ as electron donor [31]. *C. aggregans* appears, therefore, to be endowed with a metabolic ductility that makes it interesting both from an evolutionary point of view, as an archetype for studying the origin of photosynthesis, and as an untapped reservoir of catalytic activities. We report here on the characterization of a new OYE enzyme from this extremophile microorganism.

## 2. Materials and Methods

All chemicals were purchased from Sigma-Aldrich (Milano, Italy). The antibiotic Kanamycin was purchased from GeneSpin (Milano, Italy). BenchTop 1 kb DNA Ladder and Loading Dye 6X were from Promega (Milano, Italy). Gel Stain GELRED^TM^ 10000X was purchased from Biotium. Restriction enzymes, T4 DNA Ligase and Phusion^®^ High-fidelity DNA polymerase were obtained from New England Biolabs (NEB, Ipswich, MA, USA). TSAP (Thermosensitive Alkaline Phosphatase) and GoTaq^®^ DNA polymerase were purchased from Promega. Acrylamide 40% (acrylamide: bis-acrylamide ratio 37.5:1) was from PanReac AppliChem (Cinisello Balsamo, Italy). PVDF blotting membrane Amersham Hybond (0.45 μm pore size) and Amersham Hyperfilm ECL were from GE Healthcare Life Science (Milano, Italy) and SuperSignal^®^ West Pico Chemiluminescent substrate was from Thermo Fischer Scientific (Waltham, MA, USA). Anti-His-tag antibodies were purchased from Sigma-Aldrich while goat-anti-mouse antibodies labeled with peroxidase were from KPL (Milford, MA, USA). RotiMark^TM^ tricolor protein ladder (10–245 kDa) was purchased from Carl Roth (Karlsruhe, Germany). Precast NuPAGE^TM^ Tris-Acetate 3–8% gel from Thermo Fisher (Waltham, MA, USA) and SERVA native marker were for native PAGE. IMAC-Select Affinity Gel resin was purchased from Sigma-Aldrich, the Superose 12 10/300 GL columns was from GE Healthcare Life Sciences. Vivaspin^®^ centrifugal concentrators (cut off MW 10,000 kDa) were purchased from Sartorius (Varedo, Italy). SYPRO^®^ Orange Protein Gel Stain was purchased from Sigma-Aldrich. Crystallization kits and MRC plates were from Molecular Dimensions Ltd. (Sheffield, UK).

### 2.1. Sequence Analysis and Cloning

The NCBI database was used for DNA sequence analysis; searches and multiple alignments of *Ca*OYE sequence were respectively produced by programs tBLASTn and Clustal Omega.

The genomic DNA of *Chloroflexus aggregans* DSM 9485 was purchased from DSMZ culture collection and used as starting material for an initial PCR amplification of a wider segment of DNA containing the coding sequence of interest. The obtained amplimer was then used with nested primers introducing the restriction sites NdeI and BamHI at the 5′ and 3′ end of the open reading frame, respectively. Sequence of the synthetic oligonucleotides used for PCR amplifications (CaOYE1_for and CaOYE2_rev) and mutagenesis (CaOYE3_for and CaOYE4_rev) are reported in Table S1. Expression vectors were produced by digestion of pET-28a(+) (Novagen, San Diego, CA, USA) with NdeI/BamHI (New England Biolabs, NEB) and ligation of the amplified *Ca*OYE sequence.

### 2.2. Expression, Analysis and Purification of Recombinant Protein

The recombinant enzyme was expressed in *E. coli* BL21 (DE3) (Novagen, San Diego, CA, USA). Preparative cultures were carried out in 1 L LB medium containing 50 μg/mL kanamycin, at 37 °C, 180 rpm, induced at OD_600_ 0.4–0.6 by IPTG (0.2 mM), supplemented with 25 μM riboflavin and cultivated at 25 °C overnight. Cells were harvested by centrifugation, disrupted by mechanical lysis (Constant Systems Cell Disruptor OneShot; Constant Systems, Kennesaw, GA, USA). The crude extract was centrifuged (4 °C, 30 min, 18,000× *g*) to isolate soluble fractions. FMN cofactor (at 100 μM final concentration) was added to the crude extract to enhance flavination. Overexpressed protein was purified by immobilized-metal affinity chromatography (IMAC) using 5 mL Ni-NTA resin (Sigma-Aldrich). The protein was eluted in 50 mM Tris-HCl pH 8.0, 250 mM imidazole solution. Enzyme purity was checked by SDS-PAGE and immunoblotting, while the concentration was evaluated by spectrophotometric measurement of free flavin cofactor titer in a solution of thermal denatured protein, calculated as previously reported [21].

### 2.3. Native Gel and NBT Staining

Native polyacrylamide gel electrophoresis was performed using precast Tris-acetate acrylamide gel 3–8% (ThermoFisher Scientific, Waltham, MA, USA), excluding SDS and β-mercaptoethanol from buffer and sample buffer. Samples were not boiled before electrophoresis either. The running gel was composed of 25 mM Tris and 19.2 mM glycine pH 8.5. Gel was run at room temperature and a constant voltage of 150 V for 3 h. The gel was colored both with Coomassie Blue staining and by nitrotetrazolium blue chloride (NBT; 1 mg/mL in 25 mM Tris and 19.2 mM glycine pH 8.5) in the presence of 200 μM NADPH and 10 mM 2-cyclohexen-1-one.

### 2.4. Analytical Gel Filtration

Size-exclusion chromatography assays were performed with an AKTA purifier system by using a Superose^®^ 12 10/300 GL column (GE Healthcare) equilibrated with 50 mM Tris–HCl pH 8.0 and 150 mM NaCl. The flow rate for protein elution was 1 mL/min. β-Amylase (200 kDa), β,γ-globulin (158 kDa), bovine serum albumin (66 kDa) and carbonic anhydrase (29 kDa) were used as molecular mass standards. Apparent Mr values of enzymes were obtained from a graph where the elution volume (Ve) of the standard proteins was plotted against log MW.

### 2.5. Thermofluor Measurements

Apparent unfolding temperatures of the recombinant enzyme, T_m_, in standard conditions (50 mM Tris-HCl pH 8.0) and in the presence of different co-solvents (ethanol, acetonitrile and dimethyl sulfoxide) at different percentages (5–40% *v/v*), were determined using the Thermofluor method as described before [21]. *Ca*OYE purified protein was used diluted to 5 μM in 50 mM Tris-HCl buffer pH 8.0. All measurements have been performed in triplicate.

### 2.6. Activity Assay and Kinetics

*Ca*OYE activity was determined by monitoring the consumption of NADPH at 340 nm (ε = 6.22 mM^−1^ cm^−1^) using an Agilent 8453 spectrophotometer against a range of activated alkenes. In case of ketoisophorone, the assay was performed at 365 nm using a molar absorption coefficient of 3.51 mM^−1^ cm^−1^ [27]. The standard assay (100 μL) was performed at 25 °C in 50 mM Tris–HCl buffer pH 8.0 containing 100 μM NADPH and 10 mM substrate dissolved in 100% *v/v* ethanol (1% final concentration). The reaction was started by adding the enzyme to 0.2 μM final concentration. One unit of ER activity is defined as the amount of protein that reduces 1 μmol NADPH per minute. Steady-state kinetic parameters of the different substrates were determined using substrate concentrations ranging from 0 to 25 mM. Data were fitted using the Michaelis–Menten equation by the program GraphPad Prism v5.0 (GraphPad Software, San Diego, CA, USA).

### 2.7. Determination of pH Optimum

For the determination of the pH optimum, the specific activity (U/mg) was evaluated in a universal buffer of constant ionic strength (50 mM CAPS, 50 mM Tris, 50 mM MES and 50 mM AcOH) adjusted to the desired pH values (4–11) at 25 °C using either NaOH or HCl. The standard assay (100 μL) was performed using 100 μM NADPH and 10 mM 2-cyclohexen-1-one as standard substrate. Reactions were started through the addition of 0.2 μM purified enzyme and monitored over 1 min.

### 2.8. Crystallization and Data Collection

Recombinant *Ca*OYE (20 mg/mL in 50 mM Tris-HCl buffer pH 8.0) was screened by high-throughput sparse matrix crystallization trials, dispensed by Oryx8 Robot (Douglas Instruments, East Gardston, UK). MRC two-drop 96-well standard plates were adopted both in the initial screenings and following optimization steps. All the conditions were deposited and left equilibrating by vapor diffusion at 293 K. A panel of about 300 crystallization conditions were tested (PACT, LMB and MORPHEUS screens, Molecular Dimension Ltd., Sheffield, UK). Flat irregular crystals of N-terminally His_6_-tagged *Ca*OYE appeared after two weeks of incubation in multiple conditions, and the most regular ones (LMB screen n.64: 100 mM sodium/potassium phosphate, pH 6.2, containing 20% *w*/*v* PEG 4000 and 6% *v/v* MPD) were crushed and used to prepare a seeds stock for the subsequent optimization experiments. Thanks to cross-seeding methods, thicker and more regular crystals were obtained in two JCSG conditions, n.52 (100 mM Tris pH 8.5 containing 1.6 M ammonium sulphate and 200 mM lithium sulphate) and n.70 (100 mM HEPES pH 7.0 containing 1.1 M sodium malonate dibasic monohydrate and 0.5% *v/v* Jeffamine^®^ ED-2003). X-ray data gave the highest resolution for *Ca*OYE grown in n.70 JCSG. X-ray diffraction data were collected at ESRF (Grenoble, France) synchrotron radiation source (for beamlines and data collections details see Table S2).

### 2.9. Model Building and Refinement

All the diffraction data were processed and analyzed by the automated pipelines feasible at ESRF synchrotron, Grenoble. In particular, the data were integrated and scaled by EDNA Autoprocessing framework (XDS, XSCALE, Pointless, Aimless) [32]. The obtained data were further cut to appropriate resolution by running Aimless through the ccp4i2 suite [33]. The same interface was used in combination with Phenix suite for any of the subsequent steps of phasing and refinement. *Ca*OYE structure was determined by molecular replacement (PHASER software [34]), using as template a model of *Ca*OYE enzyme, built by Swiss model server (5nux structure was used as template, https://swissmodel.expasy.org, accessed on 5 April 2018). The refinement steps were carried out by Refmac5 [35] and Phenix Refine software [36]. Flavin cofactor FMN was automatically imported from Coot dictionary and fitted by ligand search run. Four molecules per asymmetric unit and roughly 50% of solvent define the crystal content. Final model was traced and fully visible from Met1 to Trp354. FMN cofactor was easily placed and clearly defined in each of the four molecules present in the asymmetric unit. Final parameters obtained for the best dataset (2.40 Å) reached Rfactor/Rfree of 0.22/0.26 (for further details see Table S2).

## 3. Results

### 3.1. Identification of New Putative CaOYE and Sequence Analysis

A hypothetical protein from *Chloroflexus aggregans* (WP_015941499.1), annotated with an OYE-like FMN binding domain, was identified by a tBLASTn search restricted to photosynthetic extremophiles, using the sequence of YqjM from *B. subtilis* (P54550) as query for thermophilic-like OYE subclass or Class III (following the most recent classification proposed by [23]). The former prey protein, that we called *Ca*OYE, was identified by displaying 49% sequence identity with the probe sequence. A Clustal Omega alignment with all other Class III enzymes pointed out the catalytic residues and the finger print motifs of thermophilic-like OYEs, as reported by [37] (Figure S1).

**Figure 1:**
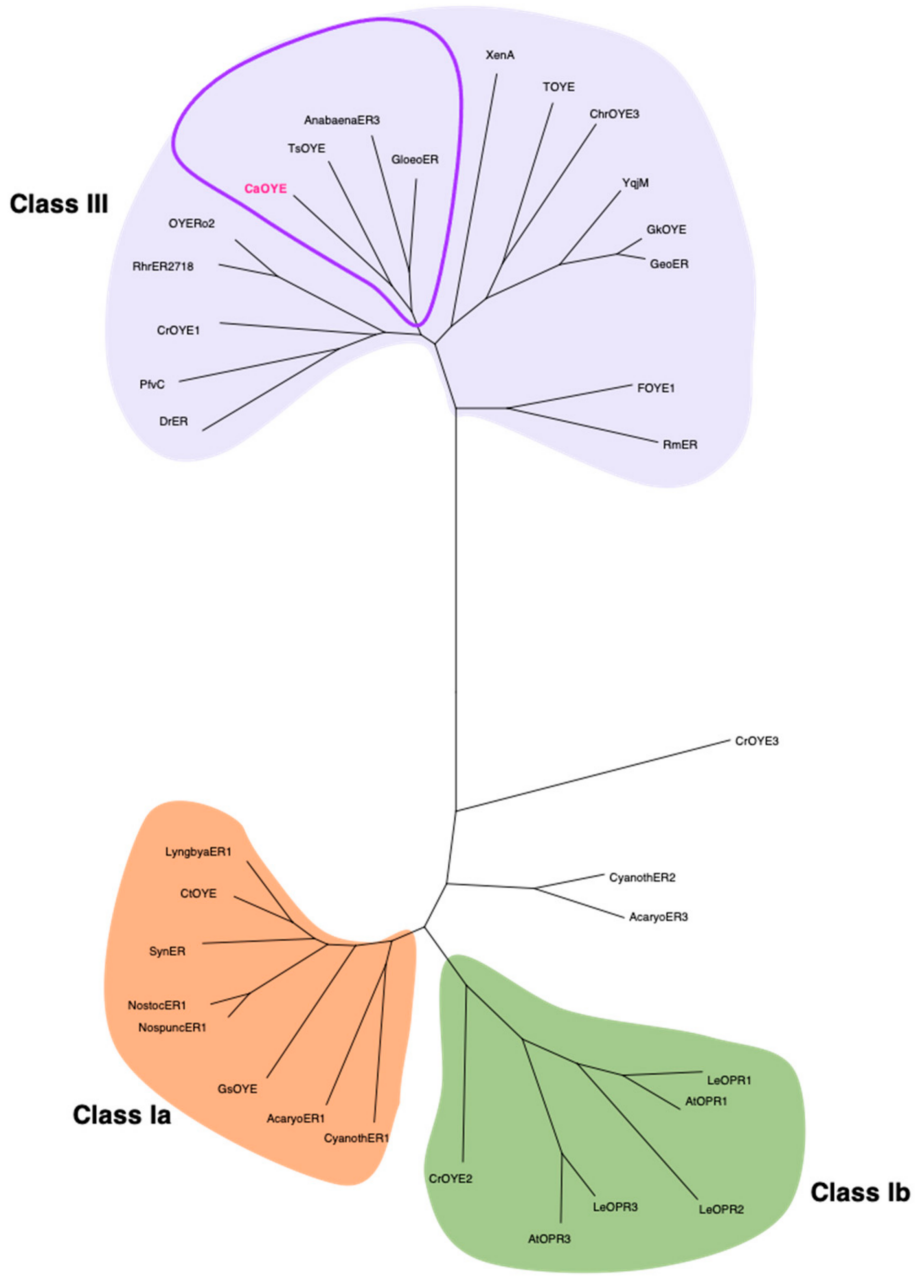
Phylogenetic analysis of *Ca*OYE and previously described OYEs from photosynthetic organisms and Class III OYEs. The evolutionary history conducted in MEGA X was inferred by using the Maximum Likelihood method. The corresponding alignment was produced via ClustalW alignment: AcaryoER1 and AcaryoER3 from *A. marina* MBIC11017, AnabaenaER3 from *T. variabilis* ATCC 29413, *At*OPR1 and *At*OPR3 from *A. thaliana*, *Ca*OYE from *C. aggregans*, *Chr*-OYE3 from *Chryseobacterium* sp. CA49, *Cr*OYE1, *Cr*OYE2 and *Cr*OYE3 from C. *reinhardtii*, *Ct*OYE from *C. thermalis*, CyanothER1 and CyanothER2 from *R. orientalis* PCC 8801, *Dr*ER from *D. radiodurans* R1, FOYE-1 from *Ferrovum* sp. JA12, *Geo*ER from *Geobacillus* sp. #30, *Gk*OYE from *G. kaustophilus* HTA426, GloeoER from *G. violaceus* PCC 7421, *Gs*OYE from *G. sulphuraria*, *Le*OPR1, *Le*OPR2 and *Le*OPR3 from *S. lycopersicum*, LyngbiaER1 from *Lyngbya* sp. PCC 8106, NospuncER1 from *N. punctiforme* PCC 73102, NostocER1 from *Nostoc* sp. PCC 7120, OYERo2 from *R. opacus* 1CP, PfvC from *Arthrobacter* sp. JBH1, *Rhr*ER2718 from *R. rhodochrous* ATCC 1795, *Rm*ER from *C. metallidurans* CH34, SynER from *S. elongatus* PCC 7942, TOYE from *T. pseudethanolicus* ATCC, *Ts*OYE from *T. scotoductus* SA-01, XenA from *P. putida*, YqjM from *B. subtilis*.

Given the increasing number of identified ene-reductases, which indeed causes an almost continuous revision of their phylogenetic classification, we chose to further compare the new sequence to a discrete number of already characterized proteins, for a total of 33 enzymes: all those known from photosynthetic organisms (cyanobacteria, algae and plants) and all the non-photosynthetic members of Class III, grouping thermostable and thermophilic-like enzymes (Table S3). In the derived dendrogram, presented in Figure 1, *Ca*OYE clustered in a sub-clade comprising the two cyanobacterial ene-reductases AnabaenaER3 and GloeoER and *Ts*OYE, from the thermophilic bacterium *Thermus scotoductus* SA-01.

### 3.2. Purification of Recombinant CaOYE and Evaluation of Its Oligomeric State

*Ca*OYE was expressed in the heterologous host *E. coli* BL21 (DE3) with an N-terminal His_6_-tag. The recombinant protein was highly over-expressed at different temperatures (18 °C, 25 °C and 30 °C); however, as a result of its massive synthesis, it partially accumulated in the form of insoluble inclusion bodies (data not shown). Nevertheless, a good amount of correctly folded protein, sufficient to perform the biochemical and biocatalytic characterization, was found in the soluble fraction; 25 °C was chosen as the best growth temperature for further purification. The protein was purified with high yields (75 mg/L) by nickel affinity chromatography, displaying a yellow color as expected for FMN binding polypeptides (Figure S2a). The Western blot analysis showed that a (lower) fraction of the recombinant product was produced as shorter, incomplete polypeptides (likely resulting from proteolysis or from abortive protein synthesis due to the presence of rare codons (16%) in the original sequence), that, however, accumulated in the insoluble fractions (Figure S2b).

The electrophoretic mobility of the recombinant *Ca*OYE was in line with the calculated molecular weight of 39.9 kDa. Considering that *Ca*OYE belongs to the thermophilic-like subclass of OYEs that generally are multimeric proteins, we decided to analyze the electrophoretic mobility of the protein also by native PAGE analysis. Again, *Ca*OYE appeared as a single band at the corresponding molecular weight of the monomeric species (Figure S2c). In order to confirm that the protein was active also in the monomeric form, we decided to stain the native gel also by nitro blue tetrazolium (NBT) (1 mg/mL) in the presence of 200 μM NADPH and 10 mM 2-cyclohexen-1-one. The NADPH consumption by *Ca*OYE lead to the precipitation of blue formazan in the gel corresponding to the native *Ca*OYE band. Furthermore, we estimated *Ca*OYE molecular weight by analytical size-exclusion chromatography and the elution volume (12.21 mL) corresponded to the attended molecular weight of monomeric *Ca*OYE, confirming the results obtained by native gel (Figure S2d). In the crystal structure of *Ca*OYE determined in this work (see Section 3.5), the typical dimeric quaternary organization of thermophilic-like OYEs has been observed in the crystal packing.

The UV-visible absorbance spectra of purified *Ca*OYE displayed maximum peak at 456 nm. Upon thermal denaturation, the supernatant turned into bright yellow and the maximum of its absorbance spectrum shifted to 446 nm (Figure S2e), corresponding to the maximum of the released free FMN. These results suggested that the flavin prosthetic group of *Ca*OYE is non-covalently bound to the protein.

### 3.3. Evaluation of CaOYE Thermal Stability and pH Optimum

**Figure 2:**
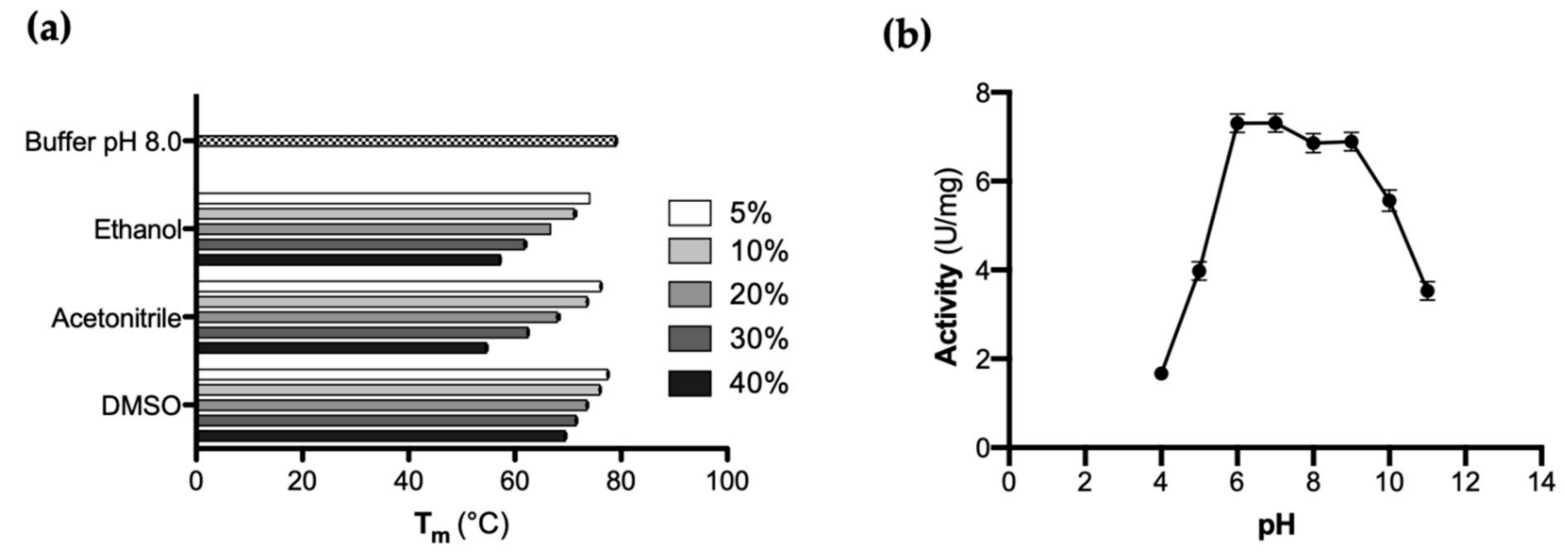
(**a**) Influence of different type and different percentages of organic co-solvents (50 mM Tris-HCl buffer pH 8.0 supplemented with increasing vol% of organic co-solvent) on *Ca*OYE protein melting point T_m_ (°C) (as measured by Thermofluor assay). All measurements have been performed in triplicate; (**b**) *Ca*OYE activity-pH profile: the specific activity (U/mg) was determined by monitoring the reduction of 2-cyclohexen-1-one in the presence of NADPH. A universal buffer of constant ionic strength, 50 mM CAPS, 50 mM Tris-HCl, 50 mM MES and 50 mM AcOH adjusted to different pH values (4.0, 5.0, 6.0, 7.0, 8.0, 9.0, 10.0 and 11.0) at 25 °C by 5 M NaOH or 5 M HCl, was used to determine the activity of the enzyme.

*Ca*OYE stability was evaluated by detecting the apparent melting temperature (T_m_) using the Thermofluor method in the presence of various co-solvents (i.e., ethanol, acetonitrile and DMSO) in different percentages. As shown in Figure 2a, the highest T_m_ for *Ca*OYE was registered in aqueous buffer at pH 8.0 (79 °C ± 0.5 °C). The enzyme well tolerated 5–10% *v/v* of ethanol, acetonitrile and DMSO but good T_m_ values were detected also in the presence of higher percentages (up to 40% *v/v*) of the same organic co-solvents (57.5 °C in ethanol, 55 °C in acetonitrile and 69.5 °C in DMSO, respectively).

Furthermore, the effect of pH on *Ca*OYE was investigated by monitoring the change in enzyme activity over a pH range of 4.0–11.0. The activity-pH profile of *Ca*OYE showed a pH optimum in the range 6.0 to 9.0, as depicted in Figure 2b. *Ca*OYE retained 50% of activity at the pH values 5.0 and 11.0, while the lowest activity was detected at pH 4.0.

### 3.4. Evaluation of CaOYE Activity with Standard Substrates and Steady-State Kinetic Parameters

A spectrophotometric assay, based on the consumption of NADPH upon reduction of some α,β-unsaturated compounds, was performed in order to explore the biocatalytic potential of the newly identified enzyme. The substrate spectrum of *Ca*OYE is summarized in Table 1: it showed very good activities with 3 out of 8 substrates tested (2-cyclohexen-1-one, 4-ketoisophorone, 1-octen-3-one) and low activities for the other substrates (from 1.1 to 2.2 U/mg). Surprisingly, *Ca*OYE displayed low activity with maleimide (2.2 U/mg), which is a generally well-accepted substrate by OYEs and high activity with ketoisophorone (8.3 U/mg). With the linear substrates tested, *Ca*OYE exhibited modest activity in the presence of 2-methyl-pentenal, while very low activity was detected in the presence of *trans*-2-hexen-1-al. 1-Octen-3-one was the preferred linear substrate for *Ca*OYE (9.4 U/mg). Steady-state kinetic parameters of *Ca*OYE with the most promising substrates detected during the spectrophotometric assay were also determined (Table 1). *Ca*OYE showed the highest catalytic efficiency with ketoisophorone (174.6 mM^−1^ s^−1^).

### 3.5. CaOYE Crystal Structure

**Figure 3:**
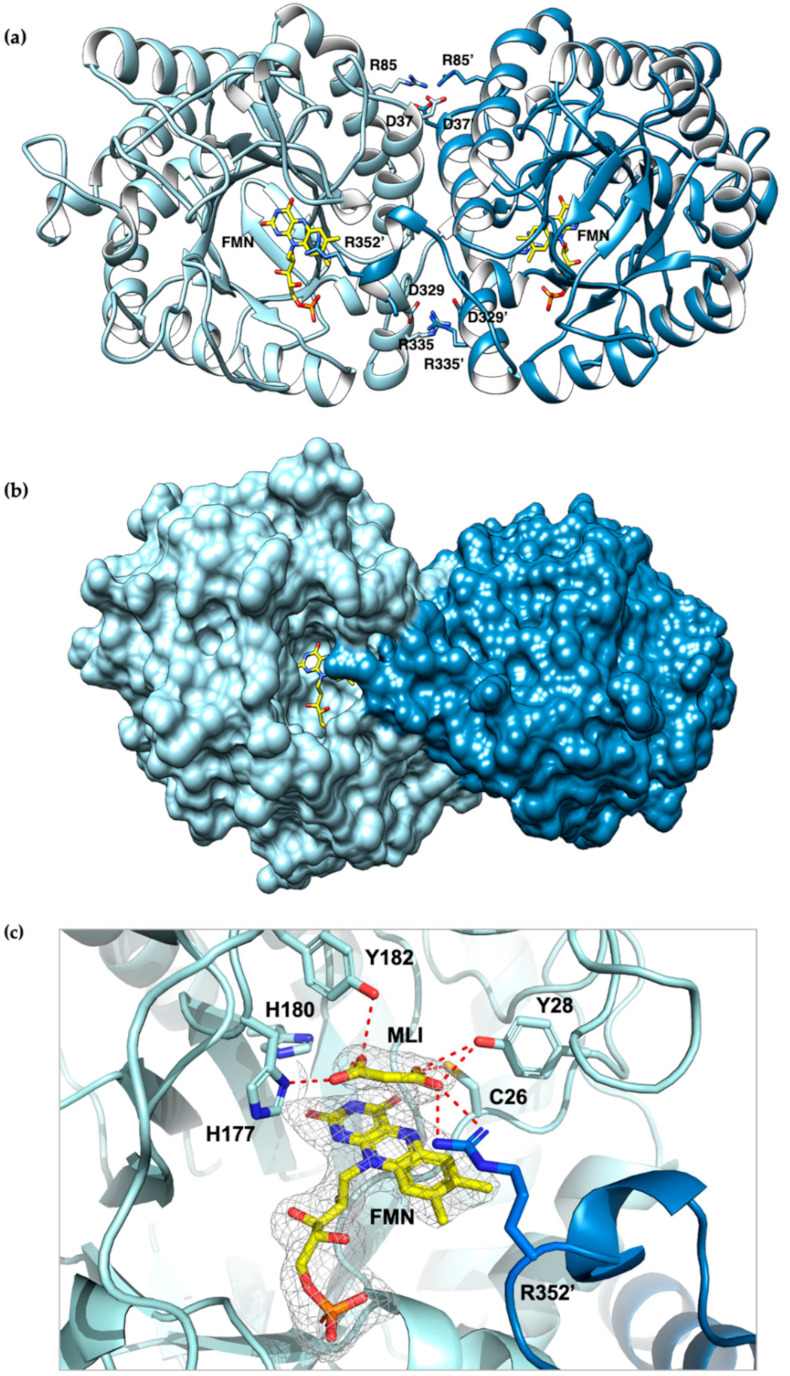
Cartoon representation of the crystal structure of *Ca*OYE (PDB 7O0T): (**a**) ribbon diagram showing the interactions involved in the dimer interface formation, (**b**) surface representation (**c**) details of *Ca*OYE active site: ligands omit map (FMN and malonate), for which the ligand residues had been omitted, has been calculated by Phenix and is shown contoured at 3 σ. The two monomers of *Ca*OYE are colored in light blue and steel blue; FMN cofactor and malonate (MLI) bound in the active site are shown with C atoms in yellow; most relevant residues of the catalytic cavity are shown with light blue C atoms, red O and blue N. Hydrogen bonds are shown as red dashed lines.

The crystal structure of *Ca*OYE (PDB 7O0T) was determined to a maximum resolution of 2.40 Å. *Ca*OYE was solved and refined in C222_1_ orthorhombic space group, with 4 molecules per ASU. The overall structure of *Ca*OYE shows the typical α_8_/β_8_ TIM barrel fold described till now, for all OYEs reported. The enzyme accommodates one molecule of FMN cofactor on the top of the β-barrel core, at the C-terminus side, buried within the active site cavity (for further details, see Figure S3). In agreement with the general behavior of Class III OYEs, crystal contacts support a dimeric architecture (Figure 3a), despite native gel analysis and size exclusion chromatography suggesting the protein behaves as a monomer in solution (Figure S2c,d, respectively). The crystallographic dimer is elongated and shows a longest axis of 94.7 Å and a mid-section of about 60.6 Å. The interaction surface between monomers in the functional dimer covers 1451 Å^2^ (Table S4). The two monomers interact via at least 8 salt bridges. Moreover, 16 hydrogen bonds and 186 hydrophobic interactions contribute to the dimerization interface (interactions calculated with the software PISA).

As already reported for the other described Class III OYEs, the C-terminus of each monomer stretches through an “Arg finger” (in our case Arg352) reaching the active site of the adjacent monomer, interacting with the respective flavin cofactor and vice versa. The *si*-side of the flavin moiety is facing a channel filled by solvent and therefore lies on the bottom of a wide-open active pocket (Figure 3b). Similar to other Class III ERs, *Ca*OYE places a conserved histidine pair (His177 and His180) in the expected positions, to be involved in the binding and correct orientation of the substrate through hydrogen bonding with the electron-withdrawing group of the substrate. The highly-conserved proton donor Tyr182 occupies the expected position, on top of the FMN flavin group, with its phenyl ring, perpendicular to the flavin plane (Figure 3c). As already mentioned, due to dimerization, the active sites are shared between the two monomers and partially shaped by the C-terminus of the neighboring subunit; in particular, Arg352 is positioned near the N5 of FMN at a distance of 5.4 Å, closing the active site (Figure 3b).

The difference electronic density map Fo–Fc shows an elongated organic molecule, most likely malonate, present within the crystallization precipitant agents, lying above the isoalloxazine ring of FMN (Figure 3c). In the complex with malonate, one carboxylate oxygen of the compound forms a hydrogen bond with the side chains of His177 but not with His180. The other carboxylate moiety of malonate is involved in a network of hydrogen bonding interactions with Tyr28, Cys25 and Arg352 of the adjacent monomer that explores the active site, suggesting a role for all these residues in substrate bonding and positioning.

## 4. Discussion

This work reports on the functional and structural characterization of a novel enzyme, the *Ca*OYE ene-reductase, having a potential interest for biocatalysis. Indeed, *Ca*OYE is being added to the over 100 ene-reductases already known, as a new member of the toolbox available for asymmetric alkene hydrogenation in industrial processes. The description of its catalytic properties and crystal structure provides new details useful for enlarging knowledge, development and application of these enzymes.

The true peculiarity of this new OYE is represented by its source, i.e., the filamentous bacterium *Chloroflexus aggregans*. This remarkable microorganism populates mats of hot springs in association with photosynthetic cyanobacteria, sulphur-oxidizing bacteria and aerobic and anaerobic heterotrophic bacteria, in micro-environmental conditions extremely dynamic in terms of light, pH, available nutrients and gases. Endowed with gliding motility, it is peculiar in the fact that it can grow photoheterotrophically, photoautotrophically, chemoheterotrophically and chemoautotrophically [30,31]. Two breakthrough works recently appeared enlarge the knowledge of *Chloroflexus aggregans* and help in better positioning it in the evolutionary scale toward the appearance of photosynthetic organisms (particularly cyanobacteria, also capable of habitat adaptation and considered the ancestors of chloroplasts). The recent descriptions of its cellular ultrastructure [38] and transcription pathways related to energy metabolism [39] highlighted astonishing morphological traits, such as a cytoplasm crowded with proto-organelles, and cycling growth modes relying on a noticeable metabolic plasticity. Likewise, it is expected that the enzymatic equipment of this organism could reveal new, interesting and original functionalities that are worthwhile to be investigated.

As previously said, our work moved from a tBLASTn search that was intentionally restricted to photosynthetic extremophiles, since we were looking for extremozymes from unusual sources. Once selected, the protein sequence from *Chloroflexus aggregans*, phylogenetic analysis indicated *Ts*OYE from the thermophilic bacterium *Thermus scotoductus* SA-01 [16], AnabaenaER3 and GloeoER, respectively from the cyanobacteria *Anabaena variabilis* ATCC 29413 and *Gloeobacter violaceus* PCC 7421 [27], as the closest homologs in a panel comprising all the so far known ene-reductases from photosynthetic organisms together with thermostable and thermophilic-like OYEs. Together with the latter enzymes, therefore, *Ca*OYE clusters in Class III of the most recent classification of OYEs [23], which identifies thermophilic and thermophilic-like enzymes.

The recombinant *Ca*OYE was shown to have a restricted substrate spectrum (with generally lower activities compared to other ene-reductases, e.g., *Ct*OYE and *Gs*OYE, [21]) and a very low activity in the presence of maleimide (which, on the contrary, is the preferred substrate of most OYEs). Notably, the same behavior was reported also for the two closely related thermophilic-like ERs AnabaenaER3 and GloeoER, isolated from cyanobacteria [27]. In this regard, it is worth noting that, similarly to *Chloroflexus aggregans*, the two cyanobacteria *Anabaena variabilis* ATCC 29413 and *Gloeobacter violaceus* PCC 7421 have long been (and still are) considered model organisms for deciphering the very first steps in the evolution of cyanobacteria. In their structural details and metabolic dynamicity, researchers looked for hints to solve fundamental questions such as the discontinuity in the passage from anoxygenic photosynthetic bacteria to oxygenic cyanobacteria [40] and the incompatibility between N_2_ fixation and O_2_ presence at the enzymatic level [41].

As for the third relative of the previously mentioned sub-clade, *Ts*OYE (see Figure 1 and further on in the Discussion), the source bacterium *Thermus scotoductus* SA-01 has also been described as a unique microorganism, whose capability to rapidly adapt to harsh environments (reducing a variety of heavy metals) stands from a hyperplastic genome [42]. It can be speculated that *Ca*OYE and its closest homologs can participate in vivo in pinpoint reactions of atypical biochemical pathways, so that standard molecules usually employed to test the biocatalytic potential of OYEs cannot mimic *Ca*OYE native substrates.

*Ca*OYE behaves as a monomer in solution while in the crystal packing assembles following the classical dimeric architecture of other thermophilic-like ene-reductases, with the peculiar C-terminal Arg finger (in our case, Arg352) of each monomer exploring the active site of the adjacent one. To our knowledge, only one thermophilic-like OYEs, i.e., *Rm*ER, has been reported to exist as monomer in solution [43]. Interestingly, *Rm*ER crystal structure has been recently solved, showing the typical dimeric assembly and interface, with the peculiar Arg finger exploring the neighboring subunit, while it loses the FMN cofactor in one of the couple active sites [44]. It has been demonstrated that the equilibrium between the different oligomeric states can be influenced by protein concentration [45]: generally, at higher protein concentration, the higher order oligomeric species are predominant, whereas at lower protein concentration, the equilibrium is shifted towards the dimeric species. Furthermore, the oligomeric state of OYERo2 was found to depend also on the interaction with NADPH and thus, on the flavin redox state. In fact, in the presence of NADPH, a full dissociation of the enzyme in homodimers was reported [45]. The equilibrium between the oligomeric forms can be concentration dependent also in the case of *Ca*OYE: the high concentrations explored by enzymes in the crystallization conditions could promote the dimeric association here described. In the attempt to deduce a correlation between the oligomeric state in solution and the main interactions observed in the crystal structures, we noticed that the percentage of the interaction surface as well as salt bridges and hydrogen bonds buried by the dimeric assembly, are the highest in *Rm*ER and *Ca*OYE, indicating that both entropic and enthalpic contributions seem to be not playing a major role in these equilibria (see Table S4).

High melting or operational temperatures were reported for *Ts*OYE (T_opt_ = 65 °C) [46], TOYE (T_m_ > 70 °C) [15], *Gk*OYE (T_m_ = 76–82 °C) [17], *Geo*ER (T_opt_ = 70 °C) [18], and FOYE-1 (T_opt_ = 50 °C) [20]. *Ca*OYE is highly thermo-resistant (T_m_ = 79 °C), with a T_m_ value comparable with that of the most thermo-resistant thermophilic-like ERs reported until now. This high thermal resistance was assigned to the extra inter-subunit interactions through hydrogen bonding and complex salt bridge networks that are formed at the dimerization interface in the quaternary structure of these enzymes. The thermal stability of *Ts*OYE was attributed to a complex salt bridges pattern defined by Asp37^#^ (^#^ from adjacent monomer), Glu85, Arg88, Arg89, and Glu92, which is responsible for the linkage between the two monomers [46]. Moreover, it has been demonstrated that the introduction of these characteristic intermolecular salt bridges in a non-thermo-tolerant OYE homologue could improve its thermal stability [45]. Analogously to *Ts*OYE, the dimer formation of *Ca*OYE is characterized by a high number of salt bridges, which could contribute to increasing its thermal stability. Further elements responsible for the thermal stability of thermophilic-like OYEs have been attributed to primary sequence content such as proline amount or Arg/Lys ratio, assuming that arginine has a higher pKa than lysine and, thus, can maintain ion pairs better at higher temperatures [46]. Indeed, in *Ts*OYE, the ratio of Arg/Lys residues is higher (4.6) compared to YqjM (0.9) and XenA (2.4). *Ca*OYE, as well, has a surprisingly high ratio of Arg/Lys residues (7.0, due to 28 Arg residues vs. 4 Lys residues) and a high content of proline residues (8.2%), that contribute to explain its high thermal resistance (T_m_ = 79 °C) (see Table S4).

In conclusion, further work will be necessary to broaden the biocatalytic spectrum and, consequently, possible applications of this new enzyme, *Ca*OYE. Nevertheless, we think that its punctual structural details, here presented, add information useful for connecting some of the dots still unlinked in the comparative analysis of the wide family of ene-reductases.

## Figures and Tables

**Table 1:** Substrate scope ^1^ and steady-state kinetic parameters ^2^ of *Ca*OYE.

Substrate		Specific Activity	Kinetic Parameters		
Structure	Name	U/mg	K_m_ (mM)	k_cat_ (s^−1^)	k_cat_/K_m_ (mM^−1^ s^−1^)
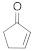	2-Cyclopenten-1-one	1.1 ± 0.09	N.D.	N.D.	N.D.
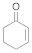	2-Cyclohexen-1-one	5.25 ± 0.22	5.520 ± 0.627	3.82 ± 0.176	0.69
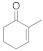	2-Methyl-cyclohexen-1-one	1.19 ± 0.07	N.D.	N.D.	N.D.
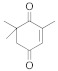	4-Ketoisophorone	8.3 ± 0.50	0.013 ± 0.002	2.27 ± 0.055	174.6
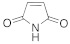	Maleimide	2.2 ± 0.37	N.D.	N.D.	N.D.
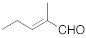	2-Methyl-pentenal	2.0 ± 0.15	N.D.	N.D.	N.D.
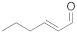	*trans*-2-Hexen-1-al	1.1 ± 0.09	N.D.	N.D.	N.D.
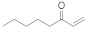	1-Octen-3-one	9.4 ± 0.40	10.32 ± 1.438	12.13 ± 0.661	1.17

^1^ The standard assay (100 μL) was performed at 25 °C in 50 mM Tris-HCl (pH 8.0) containing 100 μM NADPH and 10 mM of substrate. The reaction was started through the addition of enzyme to a final concentration of 200 nM. All measurements have been performed in triplicate. Background NADPH oxidase activity was measured in the absence of alkenes (1.0 ± 0.18 U/mg) but it was not subtracted from the specific activity with the other substrates. ^2^ The standard assay (100 μL) was performed at 25 °C in 50 mM Tris-HCl (pH 8.0) containing 100 μM NADPH, 0–25 mM of substrate and 200 nM *Ca*OYE. N.D.: not determined. All measurements have been performed in triplicate.

## Data Availability

The data presented in this study are openly available in the Protein Data Bank at www.rcsb.org with the accession code 7O0T.

## Supplementary Materials

The following are available online at https://www.mdpi.com/article/10.3390/microorganisms9050953/s1, Figure S1: Sequence alignment of the putative ER from *C. aggregans*, Figure S2: Production, oligomeric state determination and quantification of *Ca*OYE, Figure S3: Plot of FMN cofactor interactions in the active site of *holo* enzyme *Ca*OYE, Table S1: Oligonucleotides used for PCR amplification of *Ca*OYE sequences and cloning, Table S2: X-ray crystallographic data collection and refinement statistics for *Ca*OYE, Table S3: List of OYE accession numbers used for the phylogenetic analysis, Table S4: Thermostability finger prints of OYEs.
