# Supplementary material for: A New Thermophilic Ene-Reductase from the Filamentous Anoxygenic Phototrophic Bacterium Chloroflexus aggregans
Source: Microorganisms. 2021 Apr 28;9(5):953. doi: 10.3390/microorganisms9050953 (PMC8146883; doi:10.3390/microorganisms9050953)
Supplement: Supplementary file 1 [file microorganisms-09-00953-s001.zip › microorganisms-1194911-supplementary.pdf]

Microorganisms

Supplementary Material to the Article

A new thermophilic ene-reductase from the filamentous anoxygenic phototrophic bacterium *Chloroflexus aggregans*

Marina Simona Robescu[1], Mattia Niero[1], Giovanni Loprete,[1] Laura Cendron[1*], Elisabetta Bergantino[1*]

[1] Department of Biology, University of Padova, Viale G. Colombo 3, 35131 Padova, Italy

[*]Corresponding author email addresses

Elisabetta Bergantino

elisabetta.bergantino@unipd.it (+39) 0498276342

Laura Cendron

laura.cendron@unipd.it

Table of Contents

Supplementary Figures………………………………………………………………………………….…. **1**

Figure S1. Sequence alignment of the putative ER from *C. aggregans*……….. …………………………….. 1

Figure S2. Production, oligomeric state determination and quantification of *Ca*OYE…………………………..…3

Figure S3. Plot of FMN cofactor interactions in the active site of *holo* enzyme *Ca*OYE …………………….. 4

Supplementary Tables…………………………………………………………..……………………. **5**

Table S1. Oligonucleotides used for PCR amplification of *Ca*OYE sequences and cloning………………… 5

Table S2. X-ray crystallographic data collection and refinement statistics for *Ca*OYE……………………… 6

Table S3. List of OYE accession numbers used for the phylogenetic analysis………………………………. 7

Table S4. Thermostability finger prints of OYEs………………………...………………………………… 8

Supplementary Figures

```
YqjM            ---------------------------------------------MARKLFTPITIKDMT    15
XenA            ----------------------------------------------MSALFEPYTLKDVT    14
RmER            ----------------------------------------------MPHLFDPYRIGNLE    14
DrER            -------------------------------MTVSSAAAPQPASPAAPLLFTPLKLRSLE    29
RhrER2718       ----------------------------------------------VSVLFEPLTLRGVT    14
OYERo2          ----------------------------------------------MSVLFEPITFRGVT    14
PfvC            ----------------------------------------------MPALFRPLTLRSLE    14
CrOYE1          MGSATKASGSACHVPGAASTAFAQPVRKHQPMRQPLHAVGTPGPSGRPLLFEPITLRQLT    60
AnabaenaER3     -----------------------------------------------MDLFTPLIIRDIT    13
GloeoER         -----------------------------------------------MHLFAPLTLRDIT    13
CaOYE           ---------------------------------------------MQPHLFTPLTIGSVT    15
FOYE1           ----------------------------------------------MSLLFSPYQLGSLS    14
ChrOYE3         ------------------------------------------------MLYTPVNFRNVE    12
GkOYE           ---------------------------------------------MNTMLFSPYTIRGLT    15
GeoER           ---------------------------------------------METMLFSPYTIRGVT    15
TsOYE           ----------------------------------------------MALLFTPLELGGLR    14
TOYE            ----------------------------------------------MSILHMPLKIKDIT    14
                                                                 *. *  :  :

YqjM            LKNRIVMSPMCMYSSHEKD---GKLTPFHMAHYISRAIGQVGLIIVEASAVNPQGRITDQ    72
XenA            LRNRIAIPPMCQYMA-ED----GLINDWHQVHYASMARGGAGLLVVEATAVAPEGRITPG    69
RmER            LANRIAIAPMCQYSA-QE----GNATDWHMIHLGQMALSGAGLLIIEATAVSPEGRITPT    69
DrER            LPNRVVVSPMCTYSA-TD----GVANEFHLVHLGQYALGGAGLILAEATAVSPEGRITPE    84
RhrER2718       IPNRVWMAPMCQYSADVIGDQVGVPNEWHRTHLVSRAIGGTGLILTEATAVSPEGRISAA    74
OYERo2          VPNRVWMAPMCQYSADVTGRDVGVPGDWHRTHLVTRAIGGAGLILTEATAVSPEGRISPA    74
PfvC            LTHRGWVSPMCQYSCGPDG-APGVPNDWHLMHLGSFAAGGAALILTEAAAVNAAGRISPR    73
CrOYE1          IPNRIWLSPMCQYSA-VD----GAPNTWHLVHLGARAAGGCGLVMTEACAVVPEGRISPS    115
AnabaenaER3     LPSRVAMSPMCQYSA-EN----GFANDWHFVHLGSRAVGGTGLIMVEATAVTPQGRITPG    68
GloeoER         LRNRIAVSPMCQYSS-ID----GLANDWHFVHLGSRAVGGAGLVIFEAAAVEARGRISPQ    68
CaOYE           LRNRIGMSPMCQYSA-VD----GFPTDWHLMHLGARAAGGVGLIILEATAVSPEGRISPF    70
FOYE1           LANRLVIAPMCQYSA-VD----GIAQDWHLMHLGRLAISGAGLVIVEATGVNPEGRITPF    69
ChrOYE3         LKNRWVMSPMCMYSS-ED----GLPNDFHFVHYGSRAQGGAGLLIVEATGVEPRGRITNH    67
GkOYE           LKNRIVMSPMCMYSCDTKD---GAVRTWHKIHYPARAVGQVGLIIVEATGVTPQGRISER    72
GeoER           LKNRIVMSPMCMYSCDTED---GKVRTWHKIHYPTRAVGQVGLIIVEATAVTAQGRISAR    72
TsOYE           LKNRLAMSPMCQYSATLE----GEVTDWHLLHYPTRALGGVGLILVEATAVEPLGRISPY    70
TOYE            IKNRIMMSPMCMYSASTD----GMPNDWHIVHYATRAIGGVGLIMQEATAVESRGRITDH    70
                :  *  : *** * .       *    :*  *    * . .*::  ** .*   ***:

YqjM            DLGIWSDEHIEGFAKLTEQVKEQGS---KIGIQLAHAGRKAELE------------G---    114
XenA            CAGIWSDAHAQAFVPVVQAIKAAGS---VPGIQIAHAGRKASANRPWE-GDDHIG-ADDA    124
RmER            DLGLYNDANEAALGRVLGAVRNHSP--IAVTIQLAHAGRKASSEAPWD-GGGQIRPD-QP    125
DrER            DLGLWDDRQIVPLGHITDFVHQHGG---HIGVQLAHAGRKASTYAPWR-GKGAVPA--EL    138
RhrER2718       DLGIWNDTQAQAFAEINAQLAYFGA---VPGIQLAHAGRKASTQVPWR-GGKSLP-ADDR    129
OYERo2          DLGIWNDTQTEAFAEINAQLEYFGA---VPGIQLGHAGRKGSAHVPWR-GGGSLD-GDDR    129
PfvC            DAGLYNDEQAEAWQRITSFVHRHGAAEAKIGAQLAHAGRKASTYWPFSGQRGSVPESD--    131
CrOYE1          DAGMWNDAQVEAWRPIADFIRSHGA---VPSVQLAHAGRKASTFEPFVQGGRPLYPGKDA    172
AnabaenaER3     DLGLWDDKQIEPLTRIVRFLRQQGS---VTGIQLAHAGRKASCNVPWL-GGTPLTPEQ--    122
GloeoER         DLGIWSDAHIEPLRRINDFIHRQES---VAGIQIAHAGRKASTARPWE-GGGPLTEGE--    122
CaOYE           DLGIWSDDHIAALSRIVKLIESLGA---VAGIQLAHAGRKASVGRPWE-GGKPIAPAN--    124
FOYE1           CLGLYNDEQEAALGRIVAFAREFGQ--AKMAIQLAHAGRKASTRRPWD-PGSPYSPE--E    124
ChrOYE3         CMGIWNDEQAKQLQRIVEFVHKNSE--SKIGIQLSHSGRKGSTWNTK-----QIS---VE    117
GkOYE           DLGIWSDDHIAGLRELVGLVKEHGA---AIGIQLAHAGRKSQVP------------G---    114
GeoER           DLGIWSDEHVDGLRELVSLVKEHGA---KIGIQLAHAGRKAEVE------------G---    114
TsOYE           DLGIWSEDHLPGLKELARRIREAGA---VPGIQLAHAGRKAGTARPWE-GGKPL------    120
TOYE            DLGIWNDEQVKELKKIVDICKANGA---VMGIQLAHAGRKCNIS----------------    111
                  *::.: :      :                *:.*:***

YqjM            ----DIFAPSAIAFDEQSA-TPVEMSAEKVKETVQEFKQAAARAKEAGFDVIEIHAAHGY    169
XenA            RGWE-TIAPSAIAFGAHLPNVPRAMTLDDIARVKQDFVDAARRARDAGFEWIELHFAHGY    183
RmER            RGWQ-TFAPSAVPHAAGEV-PPAALDKAGMKKIRDDFVAAAKRAARLGIEGIEVHGAHGY    183
DrER            GGWQ-VIGPDENSFHDLFP-TPAMMGADELRGVVDAFSAAARRAQVAGFDAVEVHAAHGY    196
RhrER2718       LSWQ-TVAPSAVPFG-HLA-DPVELTTEGIEKVVADFAAAATRALKAEFKVVEIHAAHGY    186
OYERo2          LSWQ-TVAPSAIGFG-DHT-PPAAATTADIRKVVADFAAAAERASRAGFKVVEIHAAHGY    186
PfvC            GGWA-TVGPSPSAFD-GYA-EPAAMTEEQIQGVISDFAAAAVRAVDAGFDTLELHGAHGY    188
CrOYE1          AAWQ-VVAPSAVPFA-DFQ-TPREMTEADIREVIDAFAAAAERSIKAGFQVVEIHMAHGY    229
AnabaenaER3     GGWQ-PVAPSPIPFQENAP-VPISLDERGIQETIFAFVAAAQRALQVGFQMIEIHAAHGY    180
GloeoER         GGWADSVAPSALPFDAGYP-VPEALDEAGIAATVQAFAAAARRSLAAGFRVLEIHAAHGY    181
CaOYE           GGWP-VVGPTAEPFAPGYP-TPIPLDAAGIARVVADFATATKRARAAGFRWIEIHAAHGY    182
FOYE1           GGWQ-TWAPSAIKFYEESL-TPHPMSIEDLETVKQDFVNSAIRAERAGFKAIELHGAHGY    182
ChrOYE3         EGWE-TIAPSPIPYHPTER-IPHVLTQEEIKEQVRNFREAARRAVEAGFDVIEIHAAHGY    175
GkOYE           ----EIIAPSAVPFDDSSP-TPKEMTKADIEETVQAFQNGARRAKEAGFDVIEIHAAHGY    169
GeoER           ----EIIAPSAIPFNEKTR-TPKEMTKTDIEETIQAFQNGARRAKEAGFDVIEIHGAHGY    169
TsOYE           -GWR-VVGPSPIPFDEGYP-VPEPLDEAGMERILQAFVEGARRALRAGFQVIELHMAHGY    177
TOYE            --YEDVVGPSPIKAGDRYK-LPRELSVEEIKSIVKAFGEAAKRANLAGYDVVEIHAAHGY    168
                       .*            *       :      *   .: *:      :*:* ****
```

```
YqjM            LIHEFLSPLSNHRTDEYGGSPENRYRFLREIIDEVKQVWD--GPLFVRVSASDYTD----     223
XenA            LGQSFFSEHSNKRTDAYGGSFDNRSRFLLETLAAVREVWPENLPLTARFGVLEYDGR---     240
RmER            LLHQFLSPIANHRTDEYGGSLENRMRFPLEVFDAVREAFPAERPVWMRVSATDWVP----     239
DrER            LLHQFLSPLANTRTDDYGGSFENRTRLLLEVVRAVRHVWPAHLPLFVRLSATDWAE----     252
RhrER2718       LIHQFLSPESNKRTDRYGGSFENRIRLLLEILTAVREVWPAELPLFVRVSATDWLTEERG     246
OYERo2          LLHQFLSPVSNHRTDEYGGSFAGRIRLLLEVVDAVRGVWPAELPVFVRVSATDWLSEEPG     246
PfvC            LLHQFQSPLTNTRTDSWGGNEAGRNRLMLAVIDAVREVMPESMPLLLRISATDWAE----     244
CrOYE1          LLHQFLSPQANKRKDQYGGSLANRMRLPLAVAEAVRAVIPPELPLAVRISATDWAGP---     286
AnabaenaER3     LLHSFLSPLSNRRTDRYGGSLENRMRLLLEVVRRVRDVLPNGMPLFVRISATDWVE----     236
GloeoER         LLHSFLSPLSNRRTDRWGGLFENRIRLLLAVVEAVRGVWPERLPLFVRISATDWTE----     237
CaOYE           LLHNFLSPLGNDRNDEYGGDLRGRVRLLSEVTAAVRAEWPSDLPLAVRLSCSDWTP----     238
FOYE1           LIHQFLSPLSNQRQDQYGGSLENRMRYPLEILSAVKHALSAEMVVGMRISAVDWAP----     238
ChrOYE3         LIHQFLSPLSNIRTDEYGGSFENRIRFLVEIVDAVNQELNDNVALFVRISGTEYAE----     231
GkOYE           LINEFLSPLSNRRQDEYGGSPENRYRFLGEVIDAVREVWD--GPLFVRISASDYHP----     223
GeoER           LINEFLSPLANKRQDEYGGSSENRYRFLGEVIDAVRQVWD--GPLFVRISASDYHP----     223
TsOYE           LLSSFLSPLSNQRTDAYGGSLENRMRFPLQVAQAVREVVPRELPLFVRVSATDWGE----     233
TOYE            LIHEFLSPLSNKRKDEYGNSIENRARFLIEVIDEVRKNWPENKPIFVRVSADDYME----     224
                *  .* *   * * * :*.   .* *         *.        :  *..  ::

YqjM            ---KGLDIADHIGFAKWMKEQGVDLIDCSSGALV-HADINVFPGYQVSFAEKIR-EQADM     278
XenA            ---DEQTLEESIELARRFKAGGLDLLSVSVGFTIPETNIPWGPAFMGPIAERVR-REAKL     296
RmER            ---NGWDIEGTIALSHELKARGSAAVHVSTGGVSPQQAIKIGPGYQVPYAQRVK-AEVGL     295
DrER            ---GGWDLEQTVQLSKLLKYEGVDVLDISSGGLTAAQQIEVGPGYQVPFAAAVSRAETEI     309
RhrER2718       LEVDSWTADQTVALANILSDYGVDLVDVSTGGNSPAAQIPVEPGYQVPFARRLQ-NESLL     305
OYERo2          LDADSWTPDQTVSLVQALADLGVDLVDVSSGGVA-SARIPIGPGYQVPFARRIQ-NETTV     304
PfvC            ---GGIDIEASVRLAAQAHEHGVDLIDVSSGGAVAHQQIQPGPGYQTGFSARIR-RETRV     300
CrOYE1          LDGPAWDVDQSVVLCGALRDLGCDLIDVSSGGQLPRALIPVGPGYQVPFSERIR-REARV     345
AnabaenaER3     ---GGWDLQQSIILSRELKTLGVDLIDVSTGGLVPHARIPVEKGYQVPFAAKIR-EEAGI     292
GloeoER         ---GGWDLEQSVLLAQVLARSGVDLIDCSSGGVIPGVRIPAGPGYQTRFAERIR-AEADM     293
CaOYE           ---EGLTIADTVEVARMLREQGVDLIDCSSGGIAPGITIPVGEGYQVPFAAQVR-REANI     294
FOYE1           ---GGLTIEESITFSQECEKRGAGFIHVSTGGLVAHQQIPVGPGYQVEHAQAIK-QNVNI     294
ChrOYE3         ---NGWEITDSVELSKVLKNHSVDLVDVSSGGNIHGVKIPLFDGYQVPLASQVR-NEADV     287
GkOYE           ---DGLTAKDYVPYAKRMKEQGVDLVDVSSGAIV-PARMNVYPGYQVPFAELIR-READI     278
GeoER           ---EGLTAKDYIPYAKRMKEQGVDLIDVSSGAVV-PATINAYPGYQVPFAELIR-REAEI     278
TsOYE           ---GGWSLEDTLAFARRLKELGVDLLDCSSGGVVLRVRIPLAPGFQVPFADAVR-KRVGL     289
TOYE            ---GGINIDMMVEYINMIKD-KVDLIDVSSGGLL-NVDINLYPGYQVKYAETIK-KRCNI     278
                           :             :  * *       :    .:    :  :   .  :

YqjM            ATGAVGMITDGSMAEEILQNGRADLIFIGRELLRDPFFARTAAKQLNTEI--PAPVQYER     336
XenA            PVTSAWGFGTPQLAEAALQANQLDLVSVGRAHLADPHWAYFAAKELGVEKA-SWTLPAPY     355
RmER            PTMAVGLITEAEQAEAIIANNEADIISIARAMLYDPRWPWHAAAKLGASV--NAPKQYWR     353
DrER            SVMAVGLIETGAQAEAILQAGDADLIALGRPFLRDPHWAQRAARELGLRPV-S-IDQYAR     367
RhrER2718       PAAAVGLITEPEQAEKIVEDGSAVAVLLGRELLRDPYWARRAARELNAEVGPHIPSQYAR     365
OYERo2          PAAAVGLITEPEQAERIVESGEAVAVFLGRELLRDPYWPRKAALVLNAQVTPQIPAQYAR     364
PfvC            PTGTVGLLTSPGQAEHAVATGQADGVFIARAALRDPHWWLRAAFELGHDL--AWAPQYER     358
CrOYE1          ATGTVGLITTGKQAEKILQEGKADVVLIGREMLRDPNWPLRAAAELGYEGA-RYPPQYER     404
AnabaenaER3     MTGAVGLINEAEYADQIITRGCADLVLIGRELLRNPYWSIYARCSLDEEP--NWPVPYGY     350
GloeoER         LTGAVGQITSAEQADHIVRTGQADLVLIGRQLLRDPYWPLKAAVELRAPG--PWPEQYQR     351
CaOYE           ATAAVGLITRPEHADAIVRNGDADLVLLGRELLRDPHWPLRAARALGHDL--APPPQYLR     352
FOYE1           PTMAVGLITHSAQAETILKSEQADMIAIARAALKNPHWPWTAALELGDKP--FAPPQYQR     352
ChrOYE3         KTGAVGLIKKVSHAEEILEKGDADLIFIAREMLRNPYLAVQGSFEMNEEC--FFPHQYLR     345
GkOYE           PTGAVGLITSGWQAEEILQNGRADLVFLGRELLRNPYWPYAAARELGAKI--SAPVQYER     336
GeoER           ATGAVGLITSGWQAEEILRNGRADLVFLARELLRNPYWPYAAAKELGTTI--PAPVQYER     336
TsOYE           RTGAVGLITTPEQAETLLQAGSADLVLLGRVLLRDPYFPLRAAKALGVAP--EVPPQYQR     347
TOYE            KTSAVGLITTQELAEEILSNERADLVALGRELLRNPYWVLHTYTS-----KEDWPKQYER     333
                 . :.  :     *:  :       : :.*  * :*

YqjM            GW----------------     338
XenA            AHWLERYR----------     363
RmER            SQPRGLEKLFKDAHFGQR     371
DrER            AGW---------------     370
RhrER2718       AF----------------     367
OYERo2          AY----------------     366
PfvC            AVPRHSF-----------     365
CrOYE1          GKFPIK------------     410
AnabaenaER3     AVKRQRR-----------     357
GloeoER         AKP---------------     354
CaOYE           AW----------------     354
FOYE1           AR----------------     354
ChrOYE3         AKISS-------------     350
GkOYE           GWRF--------------     340
GeoER           GWRF--------------     340
TsOYE           GF----------------     349
TOYE            AFKK--------------     337
                .
```

Figure S1. Sequence alignment of the putative ER from *Chloroflexus aggregans* performed by Clustal Omega. The catalytic residues are highlighted in pink and the finger print motifs of thermophilic-like OYEs reported by Oberdorfer et al. (2011) are highlighted in green and cyano. The residues shaded in grey are conserved among OYE family, and the one in bold indicate similar residues. Residues highlighted in yellow show the sequence conservation of FMN binding. Residues highlighted in petrol green are involved in dimer formation. The accession numbers are reported in Table S3.

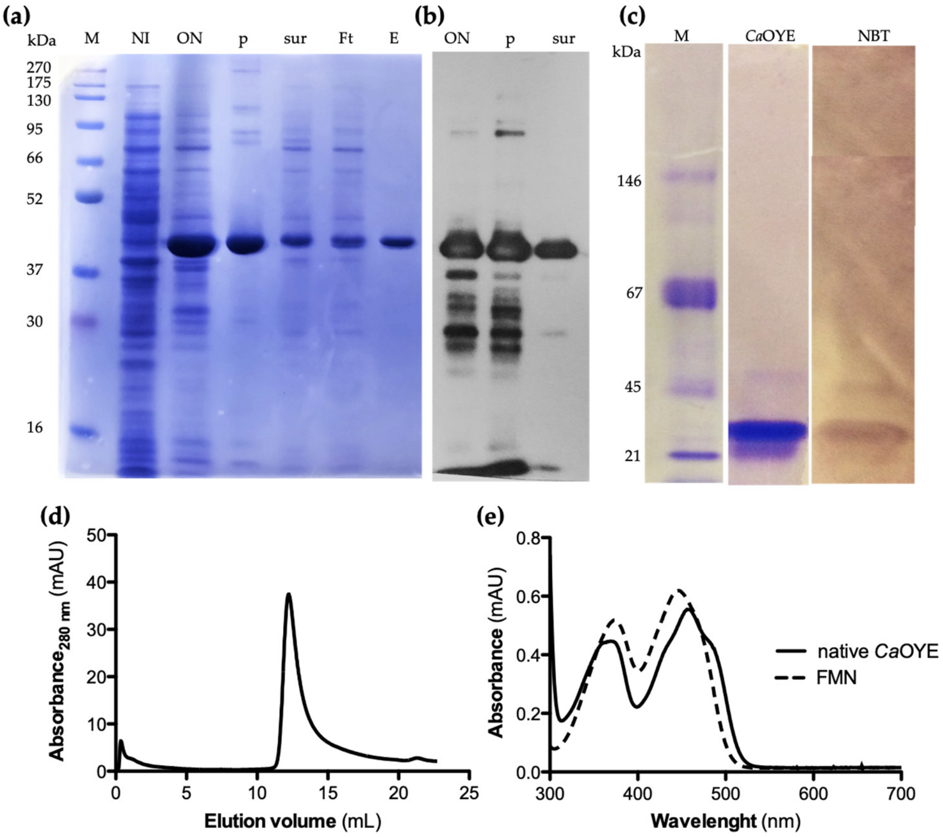

Figure S2. (**a**) SDS-PAGE 12% acrylamide: BL21(DE3) grown at 25 °C expressing *Ca*OYE: Prestained SharpMass™ VII protein ladder (M), total cell extracts from not-induced cells (NI) and overnight induced cells with IPTG (ON), pellet fraction (p), soluble protein fraction (sur), flow-through (Ft) and pooled eluted fractions from IMAC (E); (**b**) Western blot assay with anti-His tag antibodies with samples of protein induced at 25 °C; (**c**) Native-PAGE using precast NuPAGE™ Tris-Acetate 3-8% gel from ThermoFisher: SERVA native marker (M), *Ca*OYE samples stained by Commassie brilliant blue and by NTB stain; (**d**) Analytical gel filtration of purified *Ca*OYE sample eluted from a Superose 12 300/GL column.; (**e**) Determination of *Ca*OYE concentration based on flavin absorption spectra of purified enzymes and released flavin after thermal denaturation (dotted line).

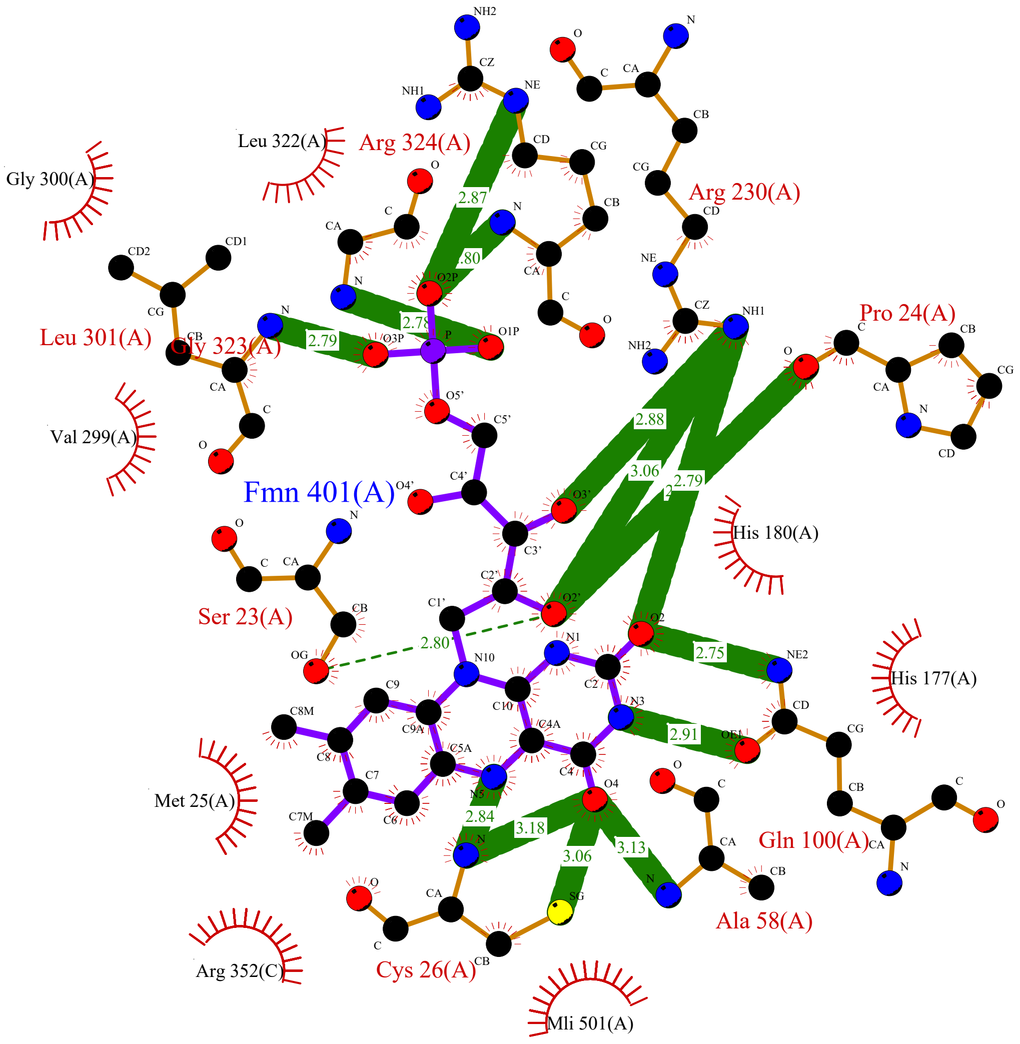

Figure S3. Schematic illustration of the interaction of FMN with active site residues of *Ca*OYE. The thin green dotted lines illustrate hydrogen bonds.

Supplementary Tables

Table S1. Oligonucleotides used for PCR amplification of *Ca*OYE sequence and cloning.

Primer	Sequence (5′→3′)[1]
CaOYE 1_for	ATGATGCAATTAAGCAGCACGTCCG
CaOYE 2_rev	GCGCGCATGACGAATTTATCGTACC
CaOYE 3_for	TTCAACTACTGAGAGAGC**<u>CAT</u>ATG**CAACCACATTTATTTAC
CaOYE 4_rev	TATA**GGATCC**TCACCACGCCCGCAAATACTGC

[1] Restriction sites are shown in bold. Modifications introduced to mutate the original sequences are underlined.

Table S2. X-ray crystallographic data collection and refinement statistics of *Ca*OYE (pdb 7O0T).

**Data collection statistics**	
Wavelength	0.97
Space group	C 2 2 21
Cell constants	
a, b, c (Å)	108.194 162.274 216.2
α, β, γ (°)	90 90 90
Resolution range for refinement (Å)	38.01 - 2.40 (2.641 - 2.40)
Total reflections	343346 (22759)
Number of Unique Reflections	73908 (4571)
R_{merge}	0.185 (0.522)
<I / sigma(I)>	4.9 (1.8)
Completeness (%)	99.3 (100.00)
Multiplicity	4.6 (5.0)
**Refinement statistics**	
R_{work} / R_{free}	0.22/0.26
RMSD bond lengths (Å)	0.0068
RMSD bond angles (°)	1.47
Average B factor	34.24
Main chain B factor	34.38
Ligands B factor	26.97
Water molecules B factor	29.45
**No. of non-hydrogen atoms**	
Number of protein atoms	10754
Number of ligands atoms	159
Number of water molecules atoms	188
**Ramachandran plot (%)**	
Most favoured	95.03
Generously allowed	4.54
Outliers	0.43

Table S3. List of OYE sequences used for the phylogenetic analysis, and their accession numbers from the National Center for Biotechnology Information (NCBI) or Phytozome (*).

Protein name	Organism	Accession number
AcaryoER1	*Acaryochloris marina* MBIC11017	ABW29811
AcaryoER3	*Acaryochloris marina* MBIC11017	ABW32756
AnabaenaER3	*Trichormus variabilis* ATCC 29413	ABA25236
*At*OPR1	*Arabidopsis thaliana*	NP_177794
*At*OPR3	*Arabidopsis thaliana*	NP_001077884
*Ca*OYE	*Chloroflexus aggregans*	WP_015941499.1
Chr-OYE3	*Cryseobacterium* sp. CA49	AHV90721
*Cr*OYE1	*Chlamydomonas reinhardtii*	Cre01.g050150*
*Cr*OYE2	*Chlamydomonas reinhardtii*	Cre03.g210513*
*Cr*OYE3	*Chlamydomonas reinhardtii*	Cre17.g727300*
*Ct*OYE	*Chroococcidiopsis thermalis*	WP_015152687.1
CyanothER1	*Rippkaea orientalis* PCC 8801	ACK64210
CyanothER2	*Rippkaea orientalis* PCC 8801	ACK65723
*Dr*ER	*Deinococcus radiodurans* R1	AAF11740
FOYE-1	*Ferrovum* sp. JA12	KRH78075
*Geo*ER	*Geobacillus* sp. #30	BAO37313
*Gk*OYE	*Geobacillus kaustophilus* HTA426	BAD76617
GloeoER	*Gloeobacter violaceus* PCC 7421	BAC91769
*Gs*OYE	*Galdieria sulphuraria*	XP_005703492.1
*Le*OPR1	*Solanum lycopersicum*	NP_001234781
*Le*OPR2	*Solanum lycopersicum*	NP_001233868
*Le*OPR3	*Solanum lycopersicum*	NP_001233873
LyngbyaER1	*Lyngbya* sp. PCC 8106	EAW37813
NospuncER1	*Nostoc punctiforme* PCC 73102	ACC84535
NostocER1	*Nostoc* sp. PCC 7120	BAB73564
OYE*Ro*2	*Rhodococcus opacus* 1CP	ALL54975
Pfvc	*Arthrobacter* sp. JBH1	AFF18622
*Rhr*ER2718	*Rhodococcus rhodochrous* ATCC 17895	AMD82542
*Rm*ER	*Cupriavidus metallidurans* CH34	ABF11721
SynER	*Synechococcus elongatus* PCC 7942	ABB56505
TOYE	*Thermoanaerobacter pseudethanolicus* ATCC	ABY93685
*Ts*OYE	*Thermus scotoductus* SA-01	CAP16804
XenA	*Pseudomonas putida*	AAF02538
YqjM	*Bacillus subtilis*	BAA12619

Table S4. Thermostability fingerprints. Parameters referring to primary sequence have been calculated with Protparam Expasy; oligomerization states in solution have been obtained from literature data and derived from size exclusion, native gel or light scattering measurements; in crystal oligomerization data were deduced from crystallographic structures, as well as interface area and number of interactions (Hydrogen bonds, Hb, and Salt bridges, Sb) involving the dimerization surface, calculated by PISA software; T_{opt} corresponds to the optimal temperature for enzymatic activity and it has been deduced from literature data, while T_m corresponds to melting temperature of the corresponding recombinant enzymes, measured either by Fluorescence (ThermoFAD or Thermofluor) or Circular dichroism.

				**Oligomerization**					
**Enzyme**	**Sequence length (aa)**	**Arg/Lys ratio**	**Total proline content (%)**	**In solution**	**In crystal**	**T_{opt} (°C)**	**T_m (°C)**	**Interface (Å², %tot)**	**Interactions (Hb, Sb)**
*Ca*OYE	354	7.0	8.2	monomer	dimer	-	79	1451 (10.4%)	(16; 8)
FOYE1	354	1.6	6.2	-	-	50 ($t_{1/2}$= 5h)	-	-	-
*Gk*OYE	340	2.2	6.2	tetramer	dimer	70	76-82	1211 (8.9%)	(16; 0)
GeoER	340	1.7	5.0	trimer or tetramer	-	70	-	-	-
*Ts*OYE	349	4.6	8.0	dimer	octamer of dimers	65	-	1273 (9.3%)	(16; 2)
TOYE	337	0.7	3.6	tetramer, octamer, dodecamer	dimer of dimers	-	> 70	1073 (8.0%)	(15; 0)
YqjM	338	0.9	3.8	tetramer	dimer of dimers	25-30 ($t_{1/2}$= 9 days)	-	1152 (8.5%)	(13; 0)
XenA	363	2.9	6.1	dimer	dimer	-	50.4	1346 (9.7%)	(2; 2)
*Rm*ER	371	2.0	7.0	monomer	dimer	35	-	2132 (14%)	(28; 7)
*Dr*ER	370	4.4	6.2	dimer	-	30	-	-	-
*Chr*OYE3	350	1.3	4.0	dimers, tetramers	-	35 ($t_{1/2}$= 233.5h)	-	-	-
*Rhr*ER2718	367	3.4	6.3	dimer	-	40	-	-	-
OYERo2	366	6.3	6.8	dimer/ tetramer conc. dependent	-	up to 20	-	-	-

PfvC	365	12.5	6.0	-	-	-	-	-	-
*Cr*OYE1	410	2.5	9.0	-	-	40 ($t_{1/2}$= 5.7 days)	-	-	-
Anabaena ER3	357	4.8	7.0	-	-	-	-	-	-
GloeoER	354	10.7	6.2	-	-	-	-	-	-
